# Invisible Wounds: Exploring the Coping Strategies of Black Survivors of Homicide Victims in Canada

**DOI:** 10.1177/00302228241246423

**Published:** 2024-04-18

**Authors:** Tanya Sharpe, Nauman Aqil, Victoria Donkin

**Affiliations:** 1 Factor-Inwentash Faculty of Social Work, 7938 University of Toronto, Toronto, ON, Canada

**Keywords:** blacks, coping, homicide, support

## Abstract

Canada has experienced a steady increase in homicide. Specifically, out of the 10 provinces and 3 territories, Ontario has consistently experienced the highest number of homicides, the majority concentrated within predominantly African, Caribbean, and Black (ACB) communities in the Greater Toronto Area (GTA). Despite this disproportionate reality, there is limited research on the ways in which survivors of homicide victims cope with the murder of their loved ones. This article explores the identification and characterization of coping strategies for ACB survivors of homicide victims residing in five neighbourhoods in the GTA. Participants in this study provided their insights and experiences, highlighting the coping mechanisms employed, the influence of cultural identity, and the challenges they experienced in accessing adequate care following the death of their loved ones. Implications for future research, policy and practice are discussed.

On average 600 murders occur in Canada each year and that number has been steadily increasing, as evidenced by the latest report from Statistics Canada, which indicates that the national homicide rate of 2.25 is the highest since 1992 (Statistics Canada, 2023b). The 8.2% increase in Canada’s homicide rate from 2021 to 2022 represents the fourth consecutive increase since 2018 (Statistics Canada, 2023b). African, Caribbean, and Black (ACB) populations comprise only 4.3% of the Canadian population (Statistics Canada, 2024) and yet the rate of homicide victims who identify as ACB is 4 times higher than the rate for non-racialized people.

Out of 13 Canadian provinces and territories the number of homicides is often highest in Ontario. Of the 288 homicides reported in Ontario, 137 victims were racialized, a proportion significantly higher than the percentage of racialized populations in Ontario (Statistics Canada, 2023d; 2023e). Out of the racialized population of homicide victims, ACB people represented over half of murdered victims (54%) and yet account for 15% of the total racialized population in Ontario (Statistics Canada, 2023e). The proportion of ACB homicide victims in Ontario is even higher than the national rate where 43% of the racialized victims are ACB (Statistics Canada, 2023a). Toronto, home to the largest number of racialized persons in Canada, recorded 136 homicides which is two fewer than the combined number of homicides reported in Montreal and Vancouver (Statistics Canada, 2023c).

Research indicates that following a homicide, approximately 7–10 family members and friends of murdered victims, (hereafter referred to as survivors), must grapple with learning how to cope with the traumatic impact of their loved one’s violent death (Gross, 2007; Rheingold et al., 2012). Moreover, Black communities often include “chosen family”, individuals who are not biologically related, suggesting that the actual number of family members coping with the murder of their loved ones may exceed current research estimates. Despite this inordinate reality, research relevant to the ways in which Black surviving family members and friends cope with the traumatic impact of experiencing the murder(s) of their loved ones is limited.

The chronic and cumulative prevalence of violent death for ACB communities highlights the importance of understanding how ACB communities are not only impacted by homicide but what barriers and facilitators exist that can best support them as the grieve and bereave their loved ones. In addition, most studies on homicide have centered on experiences in the United States of America (USA) (Connolly & Gordon, 2015). This gap in research limits the ability of Canadian policy makers, medical and mental health care providers, etc. to make informed decisions about the ways to best support surviving family member and friend of homicide victims.

## Literature Review

### Ontario’s Black Population

In 2021, Ontario was home to 768,735 Black persons, representing more than half of Canada’s Black population (Statistics Canada, 2022a). The 2021 Census data indicates that the number of Black Ontarians born outside of Canada exceeded those born within the country (Statistics Canada, 2022d). Of the 376,870 Black Ontarians born outside Canada, 115,805 migrated between 2011 and 2021 (Statistics Canada, 2022c). Regarding ethnic or cultural origins, a large proportion of Ontario’s Black population has Caribbean roots, including 166,350 Jamaicans, 38,875 African Caribbean, and 22,855 Trinidadian/Tobagonian (Statistics Canada, 2022e). Other prominent ethnic and cultural groups within the Black population in Ontario include individuals from Somalia, Nigeria, Ghana, and Ethiopia. An overwhelming majority (94.3%) of Black Canadians reside in a Census Metropolitan Area, with 442,015 individuals calling Toronto their home (Statistics Canada, 2022b).

The rich diversity within the Black population, encompassing more than 300 ethnic and cultural origins, indicates that experiencing the homicide of a loved one impacts Black communities both locally and globally. In addition, the assumption of uniformity of Black people suggests that acknowledging and understanding the diverse ways Black communities cope with the homicide of loved ones is critical to being able to provide them with culturally appropriate grief and bereavement support. It is within this context that the terms African, Caribbean, and Black (ACB) and Black will be used interchangeably throughout this paper respective of context.

## Impacts of Homicide

Experiencing the murder of a loved one has a devastating impact on survivors, affecting their physical, mental, emotional, and spiritual well-being. Compared to survivors of other violent crimes, homicide survivors are at a heightened risk of developing post-traumatic disorder (PTSD) symptoms (Currier et al., 2008; Zinzow et al., 2011). Homicide survivorship entails unique characteristics that contribute to its distinct challenges. These include exposure to media coverage, heightened scrutiny by the wider society, stigma, and discrimination in navigating the justice system, and mixed feelings of fear and revenge (Hertz et al., 2005). As a result, survivors often experience a wide range of emotions and experiences, such as anger, shock, fear, dissociation, isolation, guilt, and helplessness (Bucholz, 2002; Edwards et al., 2021). The impact of homicide survivorship involves the complex interaction of grief and trauma, which could result in complicated grief and prolonged symptoms of PTSD (Hertz et al., 2005; Laurie & Neimeyer, 2008; Sharpe & Iwamoto, 2022). The traumatic nature of the experience can lead to functional impairment (Amick-Mcmullan et al., 1991), feelings of meaninglessness (Zakarian et al., 2019), hypervigilance (Smith & Patton, 2016), emotional numbing (McDevitt-Murphy et al., 2019), and an overall diminished well-being (Rheingold et al., 2012).

The ability to ‘survive’ the homicide of a loved one depends on several factors, including but not limited to coping resources, support networks, the relationship with the deceased, personal beliefs, cultural values, and social location. The process of grieving and coping with the death is often complicated for Black survivors due to constant experiences of anti-Black racism that contribute to limited educational and employment opportunities that influence inequitable access to services (e.g., medical and mental health, legal) that could support them as they grieve their loved ones death, (Hoofnagle et al., 2020; Johnson & Hong, 2017; Unnever et al., 2021; White et al., 2021). In addition, Black communities’ long-standing history of adverse encounters with law enforcement and the criminal justice system (e.g., oversurveillance, physical violence, intimidation, mass incarceration, etc.) exacerbates the trauma experienced by survivors as they attempt to reconstruct their lives in the aftermath of homicide (Sharpe, 2015; DeVylder et al., 2020; Edwards et al., 2021). Given the enduring impact of homicide on Black survivors, understanding the coping strategies and their determinants is a necessary step toward creating culturally responsive interventions designed to support them as they grieve their loved one’s tragic death.

## Stress and Coping Theory

Coping can be defined as an effort to reduce stress in the wake of an adverse situation, event, or circumstance. Lazarus (2006) explains the psychological impact of coping with stressful events through a model that focuses on two central concepts: appraisal, that is, an individual’s evaluation of the significance of an event or situation, and coping, that is, a cognitive or behavioural effort to mitigate the demands of a stressor. According to this model, an appraisal determines individuals’ coping strategies to overcome distress (Lazarus, 1993; Wen et al., 2021). Lazarus and Folkman (1984) categorized coping strategies into problem-focused (e.g., solution-based strategies) and emotion-focused coping (e.g., expression of feelings). Once individuals appraise the level of threat a situation or an event has to their personal well-being, they select either an emotion or problem focused coping strategy to use to mitigate the traumatic impact of the stressful event.

Lazarus and Folkman’s (1984) framework is notable for its emphasis on the dynamic interplay between individuals and their environment, particularly evident during the appraisal stage and subsequent selection of coping strategies. This model underscores the subjective nature of the appraisal process, highlighting how individuals evaluate events or situations based on their distinctive qualities. Stress, as defined in this framework, and the choice of coping mechanisms are dependent on the interaction between the individual and their environment.

Building on Lazarus and Folkman’s (1984) work, researchers have attempted to explore cross-cultural variance in the appraisal of stress and coping in various contexts (Connor-Smith & Calvete, 2004; Sinha & Watson, 2007; Ward & Kennedy, 2001). However, examining the coping strategies employed by survivors of homicide victims remains scarce.

## Coping in the Aftermath of Homicide

Research on coping in the aftermath of homicide found that racialized groups use distinct coping strategies deeply rooted in cultural values and traditions (Morris & Scott, 2022; Sharpe, 2015; Yan, 2017). Sharpe’s (2015) model of coping for African American survivors of homicide victims (MCAASHV) delineates meaning-making, spiritual coping, maintaining a connection to the deceased, caring for others, concealing emotions, and collective coping as coping strategies frequently employed by Black survivors. Consistent with the stress and coping framework (Lazarus, 2006), some spiritual and collective coping strategies derive from ancestral traditions and cultural values (French et al., 2020; Giles, 2010). Moreover, the coping strategies of Black survivors often diverge significantly from those of other racial and ethnic groups, primarily due to the enduring effects of intergenerational trauma and historical racism. It is crucial to recognize that anti-Black racism persists, contributing to structural inequities, the uneven distribution of resources, and the erosion of trust in support services. This understanding is essential for comprehending the identification and utilization of coping mechanisms for Black survivors of homicide victims (Egede & Walker, 2020; Peterson & Krivo, 1993; Sharpe & Iwamoto, 2022).

Sharpe (2015) found that in the aftermath of a loved one’s murder, Black survivors of homicide victims do not simply appraise the “threat” of homicide to decide on effective coping strategies for alleviating distress, rather Black survivors engage in a racial appraisal process that considers the effects of experiencing homicide within structurally racist systems, which assign value to the experience and lead to feelings of stigma, shame, blame, and a perceived lack of justice. This ‘race-based appraisal’ influences Black survivors’ assessment, access, and utilization of coping resources through a socio-cultural lens that is designed to protect them from further harm (McGuffey & Sharpe, 2015).

A systematic review conducted by Connolly and Gordon (2015) discovered that survivors’ grieving processes were often disrupted due to a lack of transparency and accountability within the criminal justice system. The dismissive treatment of Black lives within the justice system complicates the grieving process for Black survivors as they navigate coping in the aftermath of their loved ones’ murders. The absence of comprehensive research on Black survivors of homicide victims, white populations are often used as the benchmark for comparison (Granek & Peleg-Sagy, 2015). The absence of research on Black survivors of homicide victims’ experiences contributes to a lack of culturally responsive interventions designed to prevent and mitigate traumatic injury. Moreover, experiencing the murder of a loved one leaves family members and friends of the victim with invisible “wounds”, not detectable to the naked eye but in the absence of culturally responsive interventions, are left to fester and often never heal.

The overrepresentation of ACB homicide victims in Toronto places already marginalized communities at significant risk for compromised well-being. The scarcity of research on the experiences of ACB survivors of homicide victims living in Canada leaves researchers, policymakers, and practitioners with little evidence to develop culturally responsive, evidence-based interventions that support survivors as they navigate their grief and bereavement. To address this gap, the present study explores the experiences of ACB survivors of homicide victims in Toronto, with the aim to understand the impact on their well-being, coping strategies, and unmet needs. The study draws upon data from 11 focus groups involving 26 survivors of homicide victims residing in five distinct neighbourhoods within Toronto. These neighbourhoods were deliberately selected due to consistently reporting substantially higher homicide rates compared to the citywide average.

## Methods

### Sampling Procedure and Data Collection

The study findings were based on a comprehensive analysis of 11 focus groups conducted during the summer of 2021. A semi-structured interview guide was designed to explore various aspects of the experiences of ACB survivors of homicide in Toronto, Canada. These topics encompassed understanding: (1) the impact of experiencing the homicide of loved ones, and (2) the identification and utilization of coping strategies to mitigate their traumatic grief.

This study involved a total of 26 participants representing diverse demographics. Of the participants, 53% self-identified as Black Caribbean, 27% as Black African, and 20% as Black. Most participants (60%) identified as female, with the remaining identifying as male. The age range of participants varied from 16 to 57 years, with a mean age of 30 years old. Sixty-seven percent of participants identified as Christian, 20% were Muslim, and the remaining 15% did not indicate their religious/spiritual beliefs. Socioeconomic status was also considered, with approximately two-thirds of the participants reporting an annual income of less than 30,000 CAD. Moreover, most survivors resided in neighbourhoods within the Greater Toronto Area (GTA), where homicide was endemic. Specifically, participants who resided in the five neighbourhoods (e.g., Jane & Finch, Kingston-Gallaway, Malvern, Regent Park, Rexdale) were deliberately chosen due to their consistently higher rates of reported homicides. Despite comprising less than 5% of the city’s population, these neighbourhoods account for 13.7% of the total homicides in the city.

On average, participants experienced the loss of a loved one to homicide approximately eight years before participating in this study, ranging from a minimum of one year to a maximum of 16 years. At the time of the homicide, the average age of the participants was 23 years old, with the youngest participant being 12 years old and the oldest being 52 years old. Most homicide victims were male (87%), and the average age of the victims was 22 years old, with the youngest victim being 16 years old and the oldest being 30 years old. Most participants experienced the homicide of a biological family member, whereas others experienced the homicide of a friend or family friend unrelated by blood. Additionally, more than three-fourths of the participants (77%) reported experiencing the murder of three or more loved ones, whereas 11% indicated that they had experienced the murder of one loved one.

### Data Analysis

A hybrid approach to the thematic data analysis was used to interpret the data, incorporating both inductive and deductive components. The initial stages of analysis involved line-by-line open coding and, subsequently, coding of focus group transcripts using an inductive thematic data analysis approach. At this stage, a deductive approach was introduced to investigate the possibility of generating themes based on previous research and theoretical frameworks. While inductive methods are commonly associated with qualitative research, the integration of deductive analytical strategies has proven valuable in addressing issues of generalizability and reliability (Azungah, 2018; Hays & McKibben, 2021; Pearse, 2019). Deductive qualitative analysis can effectively address research objectives that are best examined within an existing conceptual framework, as seen in the examination of coping strategies among survivors (Braun & Clarke, 2006). Various deductive approaches have been proposed to analyze qualitative data, including the template approach (Crabtree & Miller, 1992), pattern matching (Bouncken et al., 2021), and the framework method (Gale et al., 2013). However, it is essential to acknowledge that deductive analyses of qualitative data have been criticized for standardizing the data and for losing richness and creativity (Bouncken et al., 2021; Langley & Abdallah, 2015). To mitigate this concern, hybrid approaches that balance inductive and deductive analyses have been advocated for (Proudfoot, 2022; Sinkovics, 2018), including a widely used hybrid system by Fereday and Muir-Cochrane (2006).

Inspired by these approaches, we adopted an analytical strategy that allowed for “…switching back and forth between the inductively and deductively driven analysis” (Hatta et al., 2020, p. 104). To organize the focused codes and identify themes, we utilized the Model of Coping for African American Survivors of Homicide Victims (MCAASHV; Sharpe, 2015). MCAASHV is based on extant literature related to African American experiences of homicide (Neimeyer, 2000; Sharpe & Boyas, 2011; Stroebe et al., 1992) and has also been validated in follow-up studies (Morris & Scott, 2022; Sharpe et al., 2023; Sharpe & Iwamoto, 2022). The deductive process of thematic analysis facilitated by MCAASHV involved organizing the focused codes into predetermined categories and themes established in the literature. However, it also allowed for the emergence of new categories and themes to accommodate the data-driven codes. To ensure inter-rater reliability, the three authors of this manuscript independently analyzed the data. In the final analysis, deductive integration of data-driven code was accepted only when the three raters reached a consensus. Therefore, while the primary strategy remained inductive, each code was tested for compatibility with the established themes. This hybrid approach allowed us to assess the reliability of the MCAASHV in the Canadian context while allowing us to refine the model given emerging themes identified from the data.

## Findings

Survivors in this study shared their insight on how they cope with the homicide of their family members and friends, the influence of cultural identity in their coping process, and the challenges they face in accessing services following the death of their loved ones.

## Spiritual Coping and Meaning-Making

Expanding on Park’s (2010) meaning-making model and Sharpe’s (2015) model of coping for African American survivors of homicide victims, which emphasizes the importance of spiritual coping and finding meaning in traumatic experiences, study findings substantiate that for ACB people, religion and spirituality serve as significant cultural resources. They provide meaning to the interpretation of stressful events and facilitate coping with associated distress (Chatters et al., 2008; Jacob et al., 2022; Taylor et al., 2013). In line with previous homicide research involving Black American participants (Armour, 2002, 2003; Burke et al., 2011; Sharpe & Boyas, 2011), our findings reveal that many survivors turned to religion and spirituality immediately following the homicide of their loved ones. These coping strategies included personal communication with God, prayer, and attending formal places of worship.

Survivors in this study found that relying on religion and spirituality as coping resources helped ease intense feelings of grief and despair. In some instances, religion “was [the] only thing” that helped them cope after the homicide of their loved ones, and it became a “vital part of who” they are now. Several survivors in this study made “sense” out of experiencing the homicide of a loved one because of their religious beliefs. Some found a greater purpose following the loss of a loved one, while others adopted religious fatalism as a coping mechanism. For example, one survivor who lost his best friend described his belief system:

I'm Muslim. So, for us ... Just to talk about death in general, we see it as something that couldn't have been stopped. Like when somebody dies, it was their time to go... So, I feel like that was somewhat of a way for me to, I guess, cope with it or like accept it, that like we're all gonna die, we all have an expiry date.

A survivor who believed in the indissolubility of the soul stated:

So for me, how I process death is very different from how a lot of people will deal with it or a look at it. For me I believe in the soul, the spirit is still here, no matter what, and they still watch over you. I always am reminded that they are around me.

A survivor who lost her cousin said: “I can't bring him back; he’s dead, he's my ancestor now.”

While the use of religion/faith was a common coping strategy used to manage the traumatic impact of experiencing the homicide of loved ones, the use of spiritual coping was not a linear process for many survivors. Often the initial devastating impact of experiencing the homicide of a loved one did not align with their ‘orienting systems,’ that is, their core beliefs and values of themselves and others (Park, 2010; Park & Folkman, 1997). For example, the notion that God protects innocent people from tragedies did not hold true for a mother who lost her son to gun violence. This dissonance led some survivors to lose their faith, with statements like “God never existed anymore” for them. A surviving sister shared her perspective on how religious explanations of death may fall short in cases of homicide:

My beliefs were broken. Everybody loved to talk about, “Oh, he's in a better place, you know, God gained our next angel.” “How you know that?” (laughs), that's my question; you go and come back because I wanna know, “How you know?”

Another study respondent described adjusting their cognitive and emotional frameworks in response to the homicide (Park & Folkman, 1997) by trying to make meaning out of their brother’s murder, “You know what, if my brother never got shot, those guys would still be out there killing other people's kids.” This quote suggests that, for some survivors, religious coping strategies may not be their primary means of coping. Instead, they turn to meaning making to navigate their grief and find a sense of purpose in the death.

## Retaining the Emotional Bond

Maintaining a connection with the deceased is a cross-cultural coping strategy for people surviving the death of a loved one (Lalande & Bonanno, 2006; Lobar et al., 2006). In many cultures, the resting places for the dead are often chosen closer to the bereaved family, which helps maintain an emotional connection with the deceased through visits to the graveyard and mausoleums (Allam, 2019). Black survivors of homicide victims have a substantial cultural impetus to maintaining a connection with their deceased loved ones due to a shared spiritual belief in the realm of ancestral spirits that they draw upon for strength to endure stress (Baloyi & Makobe-Rabothata, 2014; Sharpe, 2015). However, the intensity and extent of the connection and the rituals designed to maintain a relationship to the deceased vary significantly across cultures (Bouc et al., 2016; Klass, 1999, 2001; Krupp & Kligfeld, 1962; Lalande & Bonanno, 2006).

Consistent with earlier studies, survivors in this study identified several rituals that allowed them to maintain a connection to their deceased loved ones (e.g., visiting the grave of the victim, acknowledging death anniversaries, preserving pictures and text messages, and arranging gatherings of friends and family to collectively recount memories of time spent with their deceased loved ones) (Sharpe, 2015; Vale-Taylor, 2009).

### Rituals, Beliefs, Practices and Activism

One respondent described wearing a keepsake to preserve the memory of his friend:

I wear Jordan's shoes, right? That's his name, so I wear Jordan's shoes. And when I wear my shoes, I think about him because, um, you know, he's the one that told me about shoes.

A survivor whose 16-year-old cousin was murdered moved back to the family house where they grew up. They describe in detail the experience of revisiting the home to maintain a connection with their deceased cousin whose room remains untouched since their untimely death:

I moved back down to the family house and, uh, this is where I get my peace with my dad and my brother. You know, this is where I get my peace because when I want, I go into his room. Nothing has been touched. Nothing. Everything is the way it's been. Not ... nothing, not his shoes, not a jacket, not a shirt. Not his bed, his pillow. Nothing has been touched in his room. However, he left it that last day is however, it is now... And his bathroom… pipes have never been turned on, the toilet's never been flushed. So, I flushed it for the first time in how many years. Um, but that's how I, that's how I get ... how I, I, I feel close to him.

A few survivors were determined to ensure that the violent act of homicide did not tarnish the memory (the name) of their loved ones. A surviving friend described these sentiments:

So if I have to, if I have to move on, if the world moves on, I have to move on, but I don't wanna be like moving on, like everyone else. I wanna make sure that his name is not remembered as someone that died with 50 shots but as someone that is a member of our community and someone that should be exonerated in our community.

A survivor who lost several friends to homicide explained how cultural identity influences the process of grieving and maintaining the connection to the deceased:

I come from a Jamaican background, um, I, uh... you know, we, we, we all... our culture deal with death, we, like you know we, we go start grieving, we grieve, but then we also celebrate the life of the person, and we try to remember the person and how the spirit of how they would live if they were alive….And we eventually let go. We're very tough people, so we, Jamaicans, you'll see like, like you know, they do live a rough life.

Some survivors engaged in community activism to maintain their connection with the deceased and to help other people cope with the homicide of their loved ones. A mother whose son was murdered stated: “I was always one to be able to speak about my son's death. Um, simply because I never wanted his name to die with him”. Maintaining a connection to the deceased was a cultural and personal imperative for many survivors. Survivors of homicide victims often found individual and collective rituals, beliefs in the spiritual realm and storytelling as essential to the grieving process and associated them with the spiritual beliefs embedded in ACB cultures.

## Collective Coping and Caring for Others

The enduring effects of systemic racism, influence what Black communities both perceive and receive as sources of support to deal with daily stressors. As a result, research has consistently illustrated Black family’s heavy reliance on informal supports (e.g., family members and friends) rather than formal support systems (e.g., mental health services) in times of crisis (Monterrosa, 2021; Myers & Speight, 2010; Sharpe, 2015). Sharpe and Boyas (2011) discovered that for African American survivors of homicide victims, select family members would often cope with their grief by caring for other family members impacted by the murder of their loved one. This form of collective coping and carrying for one another provided a known trusted relatable circle of support that helped to mitigate the impact of the traumatic grief experienced.

A mother whose daughter was murdered stated that her family members were the only ones who could hold the space for her grief:

Um, our family is small but very close, and my family is the only one that I felt comfortable that I was and still is comfortable talking with my family about it. Anyone outside of that, um, I did not feel too comfortable, like, I shun away from people because I felt that people would never understand what I've been through. And I've, and I, and I experience that with people because people like some of my coworkers [will say], “Oh, at least you have, at least you have other kids then.”

Another survivor who experienced the murder of two of his friends said that people who can relate to his background and living circumstances were the most effective in helping him manage his grief:

All of us were either the same ethnic background, so we could all kind of relate to each other. We all have like, you know, the same problems and, you know, the same things in life. So easy for us to communicate to each other 'cause we could all relate. And, um, you know, I guess just growing up where we grew up kinda, you know, you start to not get immune to this, but, you know, you understand a little bit better.

Some participants engaged in community-led violence prevention and intervention initiatives (e.g., healing circles, peace walks, youth-violence prevention programs, etc.) for survivors of homicide victims to garner both emotional and substantive financial support.

A surviving mother described her rationale for establishing a support group in her community that provided grieving mothers with emotional support regarding their grief and practical support to help with daily living expenses and funeral debt:

I saw there are so many other mothers who are worse than me. We had a mother that she lost one son. She had three when she came in this country … One died, [and] two others in jail. One now is doing 15–25 years. She's alone right now. Nobody helped her ... We are so many of us now [that] we cannot even count ... And we give that mother strength … Say we are here for you … We help [with] the funerals. We help if she has any other problems, like if she has debts … For years, she cannot be the same one. So, we have to be there for her.

## Barriers to Support

Respondents spoke candidly about factors that serve as barriers to identifying and accessing post-homicide support services (e.g., financial burden, waitlist, inability to identify culturally appropriate services).

### Challenges Accessing Services

Although collective coping has been established as a cultural precedent in Black communities (Morris & Scott, 2022; Sharpe et al., 2018), respondents in this study indicated that one of the reasons Black survivors of homicide victims rely heavily on informal supports is due to a lack of affordable culturally responsive mental and medical healthcare services, victim advocacy services and peer support groups, etc. designed to support grieving Black communities. Participants indicated that when formal supports were available, they were not affordable, and information on how to access support services was limited. One surviving mother stated:

They [victim services] are not supporting you financially and nothing and, uh, you know, morally and mentally. And then the worst part is [that] at the end, you don't even have justice.

A surviving brother expressed frustration regarding his inability to connect with a grief counsellor:

I was looking for a grief counsellor... Um, and I couldn't get in contact with any. I don’t know, maybe ‘cause I didn’t make the initiative, but I just, it was just difficult for me to get into contact with grief counsellors or anyone that, or any group that, focus[es] on victims or family members.

Survivors often found themselves so grief-stricken that they could not reach out for support. If they did, delays in response by service providers and long wait lists made them rely even more on the informal support of family and friends. One respondent remarked:

[Living in] [t]he same area is very difficult, and, uh, if you go to housing, they are asking you and tell you, you have to be in the waitlist, you have to wait for years, but you are not a normal person [being a survivor of a homicide victim].

Consistent with the findings relevant to the impact of structural racism in mental health services in North America (Alang, 2019; Misra et al., 2021), some participants alluded to the differential treatment of ACB survivors of homicide victims. These perceptions and experiences of race-based service inequity shaped survivors’ attitudes toward post-homicide services. A surviving aunt stated:

Well, I didn't really know where to go because nobody was providing us with that type of information. It was more or less grieve on your own, do what you got to do. But I [had] no support group... I'm not being prejudiced. But had we been a Caucasian family, they'd have listed out all the organizations and support groups we could have gotten at that particular time.

### Pressure to be Resilient

The lack of culturally responsive support means that ACB survivors of homicide victims are often left with the option of navigating their angst while tending to the needs of grieving family members. This is consistent with Sharpe and Boyas’s (2011) study, where African American survivors of homicide victims reported placing their well-being on the “back burner” to support family members. One participant described how their aunt went back to work immediately after the murder of her child:

She went back to her work the next day, and she cared for people, and I said to her, “Auntie, how are you caring for people? You’re not even caring for yourself, like how are you helping somebody else- as a nurse and you're not even taking care of yourself,” because as Black women we get up and go, nobody feels sorry for us. Right.

One respondent who was surviving the murders of several friends described why he felt as though he could not focus on his own grief:

That's not always possible because I gotta come back to myself and be a father, you know, I can't- I can't be two places at one time mentally. Especially when you're dealing with young kids. So, it's kind of a difficult thing to balance.

One participant experienced the murder of a loved one every year since she was 14 years old. These homicides include the murder of seven family members in one year, and the participant described years where only two people that they knew died as “great.” Despite the frequency at which she experienced the violent, traumatic death of loved ones, she described the pressure and expectation to “press on.” She referred to this concept as “the Black Woman Syndrome”:

The Black Woman Syndrome of we just get up and keep going, right? I struggle with that a lot, where, um, it's just okay, tomorrow we can head- get back to work, what are you gonna do sit and bawl every day, like until like you actually just stop one day and bawl.

Not unlike this respondent, several survivors experienced the homicide of loved ones and reported feeling numb and unable to grieve. Due to the frequent and chronic exposure to homicide, participants viewed the grieving process as a luxury they could not afford. Instead, they focused on protecting surviving family members and friends, ensuring their emotional (e.g., comfort, support) and practical needs were met (e.g., food, clothing, shelter).

## Concealment

### Avoiding Stigma

Concealment of emotions was a frequently cited coping strategy by survivors in our study and the factors leading to adopting this strategy varied across respondents. One participant described anticipated social stigma as a rationale for concealing his experience with surviving the murder of his brother:

I worked in settings where like they have no clue that I grew up in the hood, or they have no clue that I lost someone to gun violence. You know how awkward that conversation would have been, right? Um, and I, and I hide it. I don't know why I do, but I hide it.

Other survivors noted that they conceal the death of their loved ones and the emotions that surround it because “not everyone's gonna understand that experience.”

### Man Up

The concealment of emotions was also influenced by gender norms, rooted in distinct cultural socialization patterns that encourage males to be ‘strong’ and refrain from expressing their emotions (Hammond, 2012). Several participants highlighted that this gendered issue was a prominent aspect of ACB households, where male individuals were expected to embody notions of toughness (Dunbar et al., 2017). One participant attributed the emotional restraint observed in ACB communities to the historical struggles faced by their ancestors. This participant highlighted that past experiences of enslavement, abuse and oppression have shaped a cultural norm that limits the emotional expression of Black males (Dunbar et al., 2017). This finding suggests that the historical trauma experienced by ACB communities continues to influence their cultural practices and behaviors, including how emotions are expressed and perceived among Black men.

A surviving friend shared his perceptions about the culture of not “opening up” about their grief:

Like even at a young age, to a lot of parents, like African parents or Caribbean parents, like they start that, “Oh, you're a boy. You shouldn't be crying.” It starts at a young age, and it builds up. So I feel like they've, it's already in, in their heads that, oh, they have to “man up” and be tough about it, and don't show any weak sides.

Another survivor highlighted the gendered aspect of concealing emotions:

I wanted to just make sure that they [victim’s family members] were okay because I knew that this was, for men especially, this is something that's very deep, and for their emotions, I know sometimes they're not gonna open up about it. They're gonna stay within themselves.

A surviving sister encouraged her son to express his emotions when he was reluctant to have someone see him crying:

You know, your uncle [the victim] cried; he wasn't afraid to show his emotion”. You know what I mean? So, what does that mean? Like, we all have emotions, and my brother was one that showed it; he never hid it.

A surviving cousin described their belief in strengthening the family as a priority over expressing their emotions:

I literally had to be strong for everybody in my family. So, I was like that person that was trying to be strong for everybody; I never sat down and talked to anybody, wasn't able to let it out, and just drowned myself in work.

### Numbing the Pain

One survivor discussed how the prevalent victimization of Black people and the tendency to conceal emotions can be seen as a coping strategy. This strategy aims to protect Black people from the pervasive impact of chronic exposure to the traumas of systemic racism and homicide:

And like our role is to really help people recognize the principles that started us, started the foundation of the Black people, especially, you know, so for us to remember who we are, because we've lost ourselves and we've lost ourselves through the pains that we've been through, and those traumas are really playing out why we do what we do, but we don't get to sit down and understand it because we don't wanna sit down to understand the pain. We don't wanna feel it.

## No Safe Haven Here

Several participants were first or second-generation immigrants who came to Canada hoping for safety and social mobility. A surviving mother expressed how this assurance of safety and well-being in Canada could not be further from reality:

Stay in Canada and in this free country and wealthy country. They say it's the haven of the world. Canada. People think that's it's the haven of the world. But still, (laughs) there is people like us (survivors of homicide victims).

A mother whose son was murdered regretted bringing her daughter to Canada:

You know what, honestly, if we had known that this is what your kids would go through, like our generation, we would have never come to Canada because this doesn't [sic] happen to our family in Jamaica.

A surviving mother vividly described the visceral reality of the increased likelihood that Black males in her neighbourhood would be murdered and discussed her response to living in that truth:

If I was to ever find out I'm pregnant with a son, I would contemplate having an abortion 'cause I don't wanna have a Black boy in this city.” Like I don't want to have a Black son in Canada, in Toronto. My friend is like “That's sad that that's even your thought.” But I'm like; he’s either gonna go to prison or the grave.

## Discussion

This study enhances our understanding of how ACB people cope with the traumatic impact of experiencing the murder of loved ones. Through focus group interviews, we identified coping methods such as spiritual coping, meaning-making, collective coping, community activism, and emotional concealment. Participants often identified with their countries of origin rather than with a collective “Black Canadian” identity, highlighting the importance of understanding the diverse cultural nuances of being an ACB immigrant experiencing the murder of a loved one in various geographic contexts. Challenges of geographical proximity to family and friends make it difficult for surviving family members to access cultural supports, including affordable, culturally responsive victim services such as burial assistance and mental health services. Despite the lack of explicit references to racial identity, participants demonstrated racial consciousness by acknowledging broader issues within the Black community. They described the disproportionate representation of ACB people in Toronto who become homicide victims and survivors yet cope without access to such services. The impact of historical and contemporary experiences of anti-Black racism on expressions of grief is significant for ACB survivors, fostering cultural norms where stigma and shame inhibit genuine emotional expressions. This socialization often jeopardizes survivors’ well-being, impeding their grieving process and hindering the development of coping mechanisms that allow for the full expression of grief. A deeper understanding of the intersection between the traumas of anti-Black racism and homicide is crucial for supporting ACB survivors. Meaning-making and collective support emerged as prominent coping strategies. The adoption of these methods is driven by the acknowledgment that individuals with similar lived experiences are effective in supporting one another through their grief, emphasizing the importance of shared understanding and social-cultural identity in the homicide grief and bereavement process. However, these coping strategies are influenced by a lack of culturally responsive services rather than solely by cultural norms. While collective coping is a cultural precedent in ACB communities, the absence of formal support systems (e.g., mental health services) or limited awareness of available support services leads grieving survivors to rely on informal support from their families and communities. Moreover, the responsibility of caring for grieving family members often falls on a single essential family member. This support includes financial assistance for funeral expenses and essential household needs, protecting family members from harm, and vigilance to prevent retaliatory actions. Without culturally responsive services, ACB survivors are often overburdened with navigating their own grief and that of family and friends, placing them at risk for prolonged/complicated grief.

## Implications for Practice

With more than three-fourths of study participants (77%) reporting experiencing the murder of three or more loved ones, the time to develop and implement services to support Black people as they grieve and bereave their loved ones is long overdue. Providing culturally attuned services to Black communities necessitates recognizing the overrepresented and underexplored experiences of Black individuals in Canada, both as homicide victims and surviving family members and friends of those victims. In addition, we must also acknowledge that Black people are not a monolith. There is a diverse interplay between race, ethnicity, religion, cultural practice, and immigration status. These factors play a role in how ACB survivors of homicide victims perceive, identify, and utilize resources to cope with the homicide of their loved ones. Black immigrants face additional challenges, such as acculturation stress, language barriers, discrimination, and limited social support (Fanfan & Stacciarini, 2020). These stressors compound traumatic homicide related grief, emphasizing the need for cultural considerations in the development and provision of grief and bereavement services.

Helping survivors navigate their grief and bereavement process requires a nuanced and intersectional understanding, distinct from coping with a natural death. Tailored support programs, culturally responsive services, and comprehensive training for service providers are critical to addressing systemic issues perpetuating trauma in ACB communities. To effectively support ACB communities, it is essential to integrate their perspectives into comprehensive training and education of service providers. This involves using a trauma-informed approach that acknowledges the historical and contemporary systems of structural inequity that often compromise the grieving process for ACB communities. Moreover, it requires practitioners to engage in culturally relevant practices, that includes incorporating informal sources of support, and religious/spiritual practices in their continuity of care.

## Strengths and Limitations/Future Research

This study contributes to the limited empirical literature on ACB Canadian survivors of homicide victims. A rigorous hybrid data analysis approach, integrating existing theoretical frameworks with inductive and deductive methods, helped to provide a nuanced understanding of strategies used to cope with the homicide of a loved one for an under researched Black population disproportionately exposed to homicide. Results can inform the development of culturally appropriate services for ACB survivors of homicide victims. Study findings provide a foundation for a broader investigation into socio-cultural factors (e.g., race, ethnicity, culture) that influence the coping strategies of Black people throughout the global diaspora. While this study has several strengths, there are limitations. Of the 26 participants, 53% self-identified as Black Caribbean. Considering the disproportionate reality of experiencing the homicide of loved ones for Black people throughout the global diaspora, the inclusion of a more diverse sample of Black people is warranted to fully capture the distinct experience and coping strategies of ACB survivors of homicide victims. Most respondents (60%) identified as female. The inclusion of a larger sample of male survivors of homicide victims should be included in future studies on coping with homicide related grief, particularly, when we consider study findings relevant to the gendered socialization of concealing emotions for Black men. Opportunities to examine coping with homicide related grief for Black 2SLGBTQIA + populations should also be explored. Given both the frequency of homicide and the impact of acculturation on ACB communities, longitudinal studies are recommended to explore the evolution of coping strategies overtime. Additionally, assessing the availability and efficacy of culturally responsive resources among study participants in Canada and their home country would help to identify diverse coping strategies and gaps in services that can guide policy recommendations for improved support for ACB survivors within a global context.

## References

[bibr1-00302228241246423] Alang S. M. (2019). Mental health care among blacks in America: Confronting racism and constructing solutions. Health Services Research, 54(2), 346–355. 10.1111/1475-6773.13115 30687928 PMC6407345

[bibr2-00302228241246423] Allam Z. (2019). The city of the living or the dead: On the ethics and morality of land use for graveyards in a rapidly urbanised world. Land Use Policy, 87, 104037. 10.1016/j.landusepol.2019.104037

[bibr3-00302228241246423] Amick-Mcmullan A. Kilpatrick D. Resnick H. (1991). Homicide as a risk factor for PTSD among surviving family members. Behavior Modification, 15(4), 545–559. 10.1177/01454455910154005 1747092

[bibr4-00302228241246423] Armour M. (2003). Meaning making in the aftermath of homicide. Death Studies, 27(6), 519–540. 10.1080/07481180302884 12814131

[bibr5-00302228241246423] Armour M. P. (2002). Journey of family members of homicide victims: A qualitative study of their posthomicide experience. American Journal of Orthopsychiatry, 72(3), 372–382. 10.1037/0002-9432.72.3.372 15792049

[bibr6-00302228241246423] Azungah T. (2018). Qualitative research: Deductive and inductive approaches to data analysis. Qualitative Research Journal, 18(4), 383–400. 10.1108/qrj-d-18-00035

[bibr90-00302228241246423] Baloyi L. Makobe-Rabothata M. (2014). The african conception of death: A cultural implication. In Jackson L. T. B. Meiring D. Van de Vijver F. J. R. Idemoudia E. S. Gabrenya Jr W. K. (Eds.), Toward sustainable development through nurturing diversity: Proceedings from the 21st International Congress of the International Association for Cross-Cultural Psychology. 10.4087/FRDW2511

[bibr8-00302228241246423] Bouc A. Han S. H. Pennington N. (2016). “Why are they commenting on his page?”: Using Facebook profile pages to continue connections with the deceased. Computers in Human Behavior, 62, 635–643. 10.1016/j.chb.2016.04.027

[bibr9-00302228241246423] Bouncken R. B. Qiu Y. Sinkovics N. Kürsten W. (2021). Qualitative research: Extending the range with flexible pattern matching. Review of Managerial Science, 15(2), 251–273. 10.1007/s11846-021-00451-2

[bibr10-00302228241246423] Braun V. Clarke V. (2006). Using thematic analysis in psychology. Qualitative Research in Psychology, 3(2), 77–101. 10.1191/1478088706qp063oa

[bibr11-00302228241246423] Bucholz J. A. (2002). Homicide survivors: Misunderstood grievers. Routledge.

[bibr12-00302228241246423] Burke L. A. Neimeyer R. A. McDevitt-Murphy M. E. Ippolito M. R. Roberts J. M. (2011). Faith in the wake of homicide: Religious coping and bereavement distress in an African American sample. The International Journal for the Psychology of Religion, 21(4), 289–307. 10.1080/10508619.2011.607416

[bibr13-00302228241246423] Chatters L. M. Taylor R. J. Jackson J. S. Lincoln K. D. (2008). Religious coping among african americans, caribbean blacks and non-hispanic whites. Journal of Community Psychology, 36(3), 371–386. 10.1002/jcop.20202 21048887 PMC2967036

[bibr14-00302228241246423] Connolly J. Gordon R. (2015). Co-Victims of homicide: A systematic review of the literature. Trauma, Violence, and Abuse, 16(4), 494–505. 10.1177/1524838014557285 25377523

[bibr15-00302228241246423] Connor-Smith J. K. Calvete E. (2004). Cross-cultural equivalence of coping and involuntary responses to stress in Spain and the United States. Anxiety, Stress and Coping, 17(2), 163–185. 10.1080/10615800410001709412

[bibr88-00302228241246423] Crabtree B. F Miller W. L (1992). A template approach to text analysis: Developing and using codebooks. In B. F. Crabtree & W. L. Miller (Eds.). In Doing qualitative research (pp. 93–109). Newbury Park: Sage.

[bibr89-00302228241246423] Currier J Holland J. M. Coleman R. A Neimeyer R. A (2008). Bereavement following violent death: An assault on life and meaning. In Perspectives of violent death (pp. 177–202). Baywood.

[bibr18-00302228241246423] DeVylder J. Fedina L. Link B. (2020). Impact of police violence on mental health: A theoretical framework. American journal of public health, 110(11), 1704–1710. 10.2105/AJPH.2020.305874 32941068 PMC7542293

[bibr19-00302228241246423] Dunbar A. S. Leerkes E. M. Coard S. I. Supple A. J. Calkins S. (2017). An integrative conceptual model of parental racial/ethnic and emotion socialization and links to children's social-emotional development among African American families. Child Development Perspectives, 11(1), 16–22. 10.1111/cdep.12218

[bibr20-00302228241246423] Edwards T. Sharpe T. Bonomo A. Massaquoi N. (2021). Exploring research on the coping strategies of black survivors of homicide victims: A scoping review protocol. BMJ Open, 11(11), Article e049784. 10.1136/bmjopen-2021-049784 PMC8562521 34725076

[bibr21-00302228241246423] Egede L. E. Walker R. J. (2020). Structural racism, social risk factors, and Covid-19—a dangerous convergence for Black Americans. New England Journal of Medicine, 383(12), Article e77. 10.1056/NEJMp2023616 32706952 PMC7747672

[bibr22-00302228241246423] Fanfan D. Stacciarini J. M. R. (2020). Social-ecological correlates of acculturative stress among latina/o and black caribbean immigrants in the United States: A scoping review. International Journal of Intercultural Relations, 79, 211–226. 10.1016/j.ijintrel.2020.09.004

[bibr23-00302228241246423] Fereday J. Muir-Cochrane E. (2006). Demonstrating rigor using thematic analysis: A hybrid approach of inductive and deductive coding and theme development. International Journal of Qualitative Methods, 5(1), 80–92. 10.1177/160940690600500107

[bibr24-00302228241246423] French B. H. Lewis J. A. Mosley D. V. Adames H. Y. Chavez-Dueñas N. Y. Chen G. A. Neville H. A. (2020). Toward a psychological framework of radical healing in communities of color. The Counseling Psychologist, 48(1), 14–46. 10.1177/0011000019843506

[bibr25-00302228241246423] Gale N. K. Heath G. Cameron E. Rashid S. Redwood S. (2013). Using the framework method for the analysis of qualitative data in multi-disciplinary health research. BMC Medical Research Methodology, 13(1), 117–118. 10.1186/1471-2288-13-117 24047204 PMC3848812

[bibr26-00302228241246423] Giles M. S. (2010). Howard Thurman, Black spirituality, and critical race theory in higher education. The Journal of Negro Education, 79(3), 354–365.

[bibr27-00302228241246423] Granek L. Peleg-Sagy T. (2015). Representations of african Americans in the grief and mourning literature from 1998 to 2014: A systematic review. Death Studies, 39(10), 605–632. 10.1080/07481187.2015.1047059 26018864

[bibr87-00302228241246423] Gross B. (2007). Life sentence: Co-Victims of homicide. Annals of the American Psychotherapy Association, 10(3), 39–44.

[bibr28-00302228241246423] Hammond W. P. (2012). Taking it like a man: Masculine role norms as moderators of the racial discrimination–depressive symptoms association among African American men. American Journal of Public Health, 102(Suppl 2), S232–S241. 10.2105/AJPH.2011.300485 22401515 PMC3477917

[bibr29-00302228241246423] Hatta T. Narita K. Yanagihara K. Ishiguro H. Murayama T. Yokode M. (2020). Crossover mixed analysis in a convergent mixed methods design used to investigate clinical dialogues about cancer treatment in the Japanese context. Journal of Mixed Methods Research, 14(1), 84–109. 10.1177/1558689818792793

[bibr30-00302228241246423] Hays D. G. McKibben W. B. (2021). Promoting rigorous research: Generalizability and qualitative research. Journal of Counseling and Development, 99(2), 178–188. 10.1002/jcad.12365

[bibr31-00302228241246423] Hertz M. F. Prothrow-Stith D. Chery C. (2005). Homicide survivors: Research and practice implications. American Journal of Preventive Medicine, 29(5 Suppl 2), 288–295. 10.1016/j.amepre.2005.08.027 16376732

[bibr32-00302228241246423] Hoofnagle M. H. Mubang R. N. Joseph D. K. Joseph B. A. Christmas A. B. Zakrison T. L. The Equity, Quality, and Inclusion in Trauma Surgery Practice Ad Hoc Task Force of the Eastern Association for the Surgery of Trauma . (2020). Eastern association for the surgery of trauma statement on structural racism, and the deaths of george floyd, ahmaud arbery, and breonna taylor. Annals of Surgery, 272(6), 911–914, 10.1097/SLA.0000000000004430 32976286 PMC7668345

[bibr33-00302228241246423] Jacob G. Faber S. C. Faber N. Bartlett A. Ouimet A. J. Williams M. T. (2022). A systematic review of Black People coping with racism: Approaches, analysis, and empowerment. Perspectives on Psychological Science: A Journal of the Association for Psychological Science, 18(2), 392–415. 10.1177/17456916221100509 36006823 PMC10018067

[bibr34-00302228241246423] Johnson E. K. Hong S. (2017). Exposing the American dilemma: How aversive racism plays a part in homicide news reception. Howard Journal of Communications, 28(3), 297–319.

[bibr35-00302228241246423] Klass D. (1999). Developing a cross-cultural model of grief: The state of the field. OMEGA - Journal of Death and Dying Journal of Death and Dying, 39(3), 153–178, 10.2190/bdtx-cye0-hl3u-nqqw

[bibr36-00302228241246423] Klass D. (2001). Continuing bonds in the resolution of grief in Japan and North America. American Behavioral Scientist, 44(5), 742–763. 10.1177/00027640121956476

[bibr37-00302228241246423] Krupp G. R. Kligfeld B. (1962). The Bereavement Reaction a cross-cultural evaluation. Journal of Religion and Health, 1(3), 222–246. 10.1007/bf01532001

[bibr38-00302228241246423] Lalande K. M. Bonanno G. A. (2006). Culture and continuing bonds: A prospective comparison of bereavement in the United States and the people's Republic of China. Death Studies, 30(4), 303–324. 10.1080/07481180500544708 16572530

[bibr39-00302228241246423] Langley A. Abdallah C. (2015). “Templates and turns in qualitative studies of strategy and management”. In Research methods for strategic management (pp. 155–184). Routledge.

[bibr40-00302228241246423] Laurie A. Neimeyer R. A. (2008). African Americans in bereavement: Grief as a function of ethnicity. Omega, 57(2), 173–193. 10.2190/OM.57.2.d 18680889

[bibr41-00302228241246423] Lazarus R. S. (1993). Coping theory and research: Past, present, and future. Psychosomatic Medicine, 55(3), 234–247. 10.1097/00006842-199305000-00002 8346332

[bibr42-00302228241246423] Lazarus R. S. (2006). Stress and emotion: A new synthesis. Springer publishing company.

[bibr43-00302228241246423] Lazarus R. S. Folkman S. (1984). Stress, appraisal, and coping. Springer Publishing Company.

[bibr44-00302228241246423] Lobar S. L. Youngblut J. M. Brooten D. (2006). Cross-cultural beliefs, ceremonies, and rituals surrounding death of a loved one. Pediatric Nursing, 32(1), 44–50. 16572538

[bibr45-00302228241246423] McDevitt-Murphy M. E. Zakarian R. J. Luciano M. T. Olin C. C. Mazzulo N. N. Neimeyer R. A. (2019). Alcohol use and coping in a cross-sectional study of African American homicide survivors. Journal of Ethnicity in Substance Abuse, 20(1), 135–150. 10.1080/15332640.2019.1598905 31044649 PMC6824931

[bibr46-00302228241246423] McGuffey C. Sharpe T. L. (2015). Racial appraisal: An integrated cultural and structural response to African American experiences with violent trauma. Journal of Sociology and Social Welfare, 3(2), 55–61. 10.15640/jssw.v3n2a6

[bibr47-00302228241246423] Misra S. Jackson V. W. Chong J. Choe K. Tay C. Wong J. Yang L. H. (2021). Systematic review of cultural aspects of stigma and mental illness among racial and ethnic minority groups in the United States: Implications for interventions. American Journal of Community Psychology, 68(3-4), 486–512. 10.1002/ajcp.12516 33811676

[bibr48-00302228241246423] Monterrosa A. E. (2021). How race and gender stereotypes influence help-seeking for intimate partner violence. Journal of Interpersonal Violence, 36(17-18), NP9153–NP9174, 10.1177/0886260519853403 31189417

[bibr49-00302228241246423] Morris K. G. Scott J. C. (2022). “No one can feel my pain”: The experience of black women survivors of homicide victims and system agents’ perspectives on the impact of homicide in the Boston area. Homicide Studies, 10.1177/10887679221139500

[bibr50-00302228241246423] Myers L. J. Speight S. L. (2010). Reframing mental health and psychological well-being among persons of African descent: Africana/Black psychology meeting the challenges of fractured social and cultural realities. Journal of Pan African Studies, 3(8), 66–82.

[bibr51-00302228241246423] Neimeyer R. A. (2000). Searching for the meaning of meaning: Grief therapy and the process of reconstruction. Death Studies, 24(6), 541–558. 10.1080/07481180050121480 11503667

[bibr52-00302228241246423] Park C. L. (2010). Making sense of the meaning literature: An integrative review of meaning making and its effects on adjustment to stressful life events. Psychological Bulletin, 136(2), 257–301. 10.1037/a0018301 20192563

[bibr53-00302228241246423] Park C. L. Folkman S. (1997). Meaning in the context of stress and coping. Review of General Psychologyf General Psychology, 1(2), 115–144, 10.1037/1089-2680.1.2.115

[bibr54-00302228241246423] Pearse N. (2019). An illustration of a deductive pattern matching procedure in qualitative leadership research. Electronic Journal of Business Research Methods, 17(3), 143–154, 10.34190/jbrm.17.3.004

[bibr55-00302228241246423] Peterson R. D. Krivo L. J. (1993). Racial segregation and black urban homicide. Social Forces, 71(4), 1001–1026. 10.2307/2580128

[bibr56-00302228241246423] Proudfoot K. (2022). Inductive/Deductive hybrid thematic analysis in mixed method research. Journal of Mixed Methods Research, 17(3), Article 15586898221126816.

[bibr57-00302228241246423] Rheingold A. A. Zinzow H. Hawkins A. Saunders B. E. Kilpatrick D. G. (2012). Prevalence and mental health outcomes of homicide survivors in a representative US sample of adolescents: Data from the 2005 National Survey of Adolescents. The Journal of Child Psychology and Psychiatry and Allied Disciplines, 53(6), 687–694. 10.1111/j.1469-7610.2011.02491.x 22211367 PMC3326225

[bibr58-00302228241246423] Sharpe T. L. (2015). Understanding the sociocultural context of coping for african American family members of homicide victims: A conceptual model. Trauma, Violence, and Abuse, 16(1), 48–59. 10.1177/1524838013515760 24370631

[bibr59-00302228241246423] Sharpe T. L. Boyas J. (2011). We fall down: The African American experience of coping with the homicide of a loved one. Journal of Black Studies, 42(6), 855–873. 10.1177/0021934710377613 22073426

[bibr60-00302228241246423] Sharpe T. L. Iwamoto D. K. (2022). Psychosocial aspects of coping that predict PTSD for african American survivors of homicide victims. Preventive Medicine: Special Issue on the Epidemiology and Prevention of Gun Violence, 165(Pt A), 107277. 10.1016/j.ypmed.2022.107277 36162488

[bibr61-00302228241246423] Sharpe T. L. Iwamoto D. K. Lee K. A. Anderson J. M. (2023). Development and validation of the inventory of stress & coping for african American survivors of homicide victims. Psychological Trauma: Theory, Research, Practice and Policy 15(5), 791–799. 10.1037/tra0001246 35511537

[bibr62-00302228241246423] Sharpe T. L. Iwamoto D. K. Massey J. M. Murphy Michalopoulos L. (2018). The development of a culturally adapted pilot intervention for african American family members of homicide victims: A preliminary report. Violence and Victims, 33(4), 708–720. 10.1891/0886-6708.VV-D-17-00052 30567769

[bibr63-00302228241246423] Sinha B. K. Watson D. C. (2007). Stress, coping and psychological illness: A cross-cultural study. International Journal of Stress Management, 14(4), 386–397. 10.1037/1072-5245.14.4.386

[bibr64-00302228241246423] Sinkovics N. (2018). “Pattern matching in qualitative analysis”. In Cassell C Cunliffe AL Grandy G (Eds.), The Sage handbook of qualitative business and management research methods: Methods and challenges (pp. 468–484). Cassell & Co.

[bibr65-00302228241246423] Smith J. R. Patton D. U. (2016). Posttraumatic stress symptoms in context: Examining trauma responses to violent exposures and homicide death among Black males in urban neighborhoods. American Journal of Orthopsychiatry, 86(2), 212–223. 10.1037/ort0000101 26963344

[bibr66-00302228241246423] Statistics Canada (2022a). Table 98-10-0308-01 visible minority by immigrant status and period of immigration: Canada, provinces and territories, census metropolitan areas and census agglomerations with parts. 10.25318/9810030801-eng

[bibr67-00302228241246423] Statistics Canada (2022b). Table 98-10-0326-01 visible minority by place of birth and generation status: Canada, provinces and territories, census metropolitan areas and census agglomerations with parts. 10.25318/9810032601-eng

[bibr68-00302228241246423] Statistics Canada (2022c). Table 98-10-0337-01 visible minority by ethnic or cultural origin: Canada, provinces and territories, census metropolitan areas and census agglomerations with parts. 10.25318/9810033701-eng

[bibr69-00302228241246423] Statistics Canada (2022d). Focus on geography series, 2021 census of population. Retrieved February 3, 2024 from. https://www12.statcan.gc.ca/census-recensement/2021/as-sa/fogs-spg/page.cfm?topic=10&lang=E&dguid=2021A000011124

[bibr70-00302228241246423] Statistics Canada (2022e). More than half of Canada’s Black population calls Ontario home. Retrieved May 27, 2023 from. https://www.statcan.gc.ca/o1/en/plus/441-more-half-canadas-black-population-calls-ontario-home

[bibr71-00302228241246423] Statistics Canada (2023a). Homicide trends in Canada, 2022. https://www150.statcan.gc.ca/n1/daily-quotidien/231129/dq231129b-eng.htm

[bibr72-00302228241246423] Statistics Canada (2023b). Table 35-10-0068-01 Number, rate and percentage changes in rates of homicide victims. 10.25318/3510006801-eng

[bibr73-00302228241246423] Statistics Canada (2023c). Table 35-10-0071-01 number and rate of homicide victims, by census metropolitan areas. 10.25318/3510007101-eng

[bibr74-00302228241246423] Statistics Canada (2023d). Table 35-10-0206-01 Number, percentage and rate of homicide victims, by racialized identity group, gender and region. 10.25318/3510020601-eng

[bibr75-00302228241246423] Statistics Canada (2023e). Table 98-10-0351-01 visible minority by gender and age: Canada, provinces and territories. 10.25318/9810035101-eng

[bibr76-00302228241246423] Statistics Canada (2024). Black history month 2023... By the numbers. https://www.statcan.gc.ca/en/dai/smr08/2023/smr08_270

[bibr77-00302228241246423] Stroebe M. Gergen M. M. Gergen K. J. Stroebe W. (1992). Broken hearts or broken bonds: Love and death in historical perspective. American Psychologist, 47(10), 1205–1212. 10.1037//0003-066x.47.10.1205 1443859

[bibr78-00302228241246423] Taylor L. K. Lasky H. L. Weist M. D. (2013). Adjusting intervention acuity in school mental health: Perceiving trauma through the lens of cultural competence. In Handbook of culturally responsive school mental health: Advancing research, training, practice, and policy. (pp. 235–250). Springer.

[bibr79-00302228241246423] Unnever J. D. Stults B. J. Messner S. F. (2021). Structural racism and criminal violence: An analysis of state-level variation in homicide. Race and Justice, 13(4), 433–462. 10.1177/21533687211015287

[bibr80-00302228241246423] Vale-Taylor P. (2009). “We will remember them”: A mixed-method study to explore which post- funeral remembrance activities are most significant and important to bereaved people living with loss, and why those particular activities are chosen. Palliative Medicine, 23(6), 537–544. 10.1177/0269216309103803 19304810

[bibr81-00302228241246423] Ward C. Kennedy A. (2001). Coping with cross-cultural transition. Journal of Cross-Cultural Psychology, 32(5), 636–642. 10.1177/0022022101032005007

[bibr82-00302228241246423] Wen X. Wang D. Li N. Qin X. Gu D. (2021). The construction of the structural equation model of burden, benefit finding, and anxiety-depression of esophageal cancer caregivers based on Lazarus stress and coping theory. Annals of Palliative Medicine, 10(7), 7644–7652. 10.21037/apm-21-1466 34353052

[bibr83-00302228241246423] White K. Stuart F. Morrissey S. L. (2021). Whose lives matter? Race, space, and the devaluation of homicide victims in minority communities. Sociology of Race and Ethnicity, 7(3), 333–349. 10.1177/2332649220948184

[bibr84-00302228241246423] Yan K. (2017). Chinese international students’ stressors and coping strategies in the United States. Springer.

[bibr85-00302228241246423] Zakarian R. J. McDevitt-Murphy M. E. Bellet B. W. Neimeyer R. A. Burke L. A. (2019). Relations among meaning making, PTSD, and complicated grief following homicide loss. Journal of Loss and Trauma, 24(3), 279–291. 10.1080/15325024.2019.1565111

[bibr86-00302228241246423] Zinzow H. M. Rheingold A. A. Byczkiewicz M. Saunders B. E. Kilpatrick D. G. (2011). Examining posttraumatic stress symptoms in a national sample of homicide survivors: Prevalence and comparison to other violence victims. Journal of Traumatic Stress, 24(6), 743–746. 10.1002/jts.20692 22108895

